# Host Single Nucleotide Polymorphisms Modulating Influenza A Virus Disease in Humans

**DOI:** 10.3390/pathogens8040168

**Published:** 2019-09-30

**Authors:** Aitor Nogales, Marta L. DeDiego

**Affiliations:** 1 Center for Animal Health Research, INIA-CISA, 28130 Madrid, Spain; 2 Department of Molecular and Cell Biology, Centro Nacional de Biotecnología (CNB-CSIC), Campus Universidad Autónoma de Madrid, 28049 Madrid, Spain

**Keywords:** Influenza A virus (IAV), innate immunity, single nucleotide polymorphisms (SNPs), Influenza vaccine

## Abstract

A large number of human genes associated with viral infections contain single nucleotide polymorphisms (SNPs), which represent a genetic variation caused by the change of a single nucleotide in the DNA sequence. SNPs are located in coding or non-coding genomic regions and can affect gene expression or protein function by different mechanisms. Furthermore, they have been linked to multiple human diseases, highlighting their medical relevance. Therefore, the identification and analysis of this kind of polymorphisms in the human genome has gained high importance in the research community, and an increasing number of studies have been published during the last years. As a consequence of this exhaustive exploration, an association between the presence of some specific SNPs and the susceptibility or severity of many infectious diseases in some risk population groups has been found. In this review, we discuss the relevance of SNPs that are important to understand the pathology derived from influenza A virus (IAV) infections in humans and the susceptibility of some individuals to suffer more severe symptoms. We also discuss the importance of SNPs for IAV vaccine effectiveness.

## 1. Introduction

### 1.1. Influenza A Virus (IAV)

**Figure 1:**
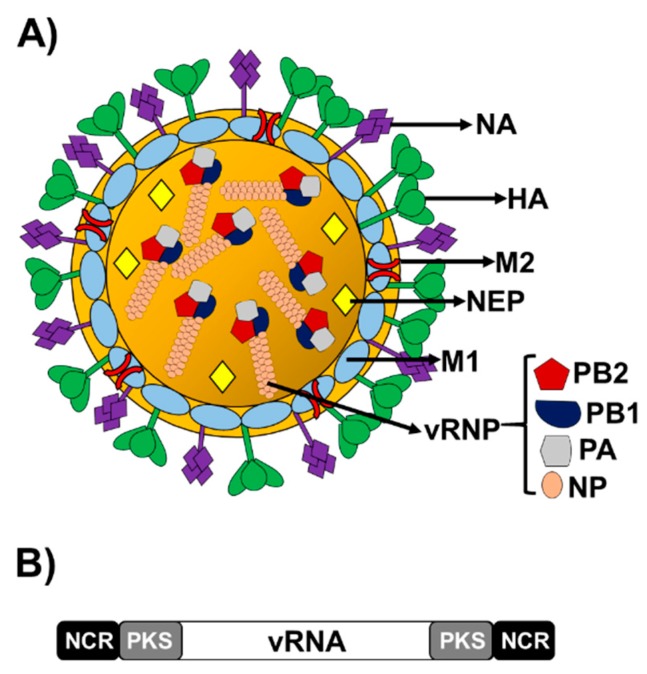
Influenza A virus structure and genome organization. (**A**) Virion structure: Influenza A viruses include a lipid envelope containing the two viral glycoproteins (HA, green and NA, purple). The ion channel M2 (red) protein is also located in the membrane. Under the viral bilayer is located a protein layer composed of the M1 (light blue) protein, and the NEP (yellow). Inside the virion are located the eight vRNA segments coated by the NP (pink) as viral vRNP complexes and associated with the viral polymerase complex made of the three polymerase subunits PB2 (red), PB1 (blue) and PA (gray). Viral components in the vRNP and in the viral particle are indicated. (**B**) Genome organization: Influenza A virus contains eight ss, negative-sense, viral RNA segments (PB2, PB1, PA, HA, NP, NA, M, and NS). Each viral segment contains non-coding regions (NCR, black) and the packaging signals (PKS, gray) at the 3′ and 5′ termini in each of the viral segments.

Influenza A viruses (IAV) belong to the *Orthomyxoviridae* family, and they contain a single-stranded (ss) negative-sense viral (v)RNA genome formed by eight segments that are encapsidated into particles with an envelope (Figure 1A). Each of the vRNA segments contains a long central coding region flanked at 5′ and 3′ termini by non-coding regions (NCRs), which work as promoters to initiate viral RNA synthesis (transcription and replication). Moreover, the packaging signals playing a role in the efficient encapsidation of the viral segments into nascent virions, are located at the 3′ and 5′ end of the coding regions (Figure 1B) [1]. Structurally, vRNAs form viral ribonucleoprotein complexes (vRNPs), where vRNAs are coated with multiple subunits of the viral nucleoprotein (NP) and are associated with the heterotrimeric polymerase, which contains the polymerase basic 2 and 1 (PB2 and PB1, respectively) and acidic (PA) proteins (Figure 1A) [2,3,4]. Each vRNP acts as an independent transcription-replication unit using an uncommon mechanism among negative-sense RNA viruses, given that viral RNA synthesis occurs in the infected-cells nucleus. vRNAs are used as templates by the viral polymerase to synthesize two positive-sense RNA molecules, the complementary RNAs (cRNAs), from which the same viral polymerase synthesizes more copies of genomic vRNA, and the mRNAs for viral protein synthesis [1,2,3,4,5,6]. The small IAV genome encodes for up to 16 viral proteins through multiple mechanisms, although there are some differences between strains. For that genome plasticity, IAV take advantage of multiple strategies, such as alternative splicing, frameshift mechanisms, and overlapping open reading frames (ORFs) [7,8,9]. In addition, the coding capability of the viral genome is extended by encoding multifunctional proteins that act at different steps during virus infection.

The viral envelope is decorated with the two viral glycoproteins hemagglutinin (HA) and neuraminidase (NA) at a ratio of approximately four to one, respectively [10,11]. HA envelope protein mediates virus entry by binding to sialic acid-containing cell receptors, and then fusing endosomal and viral membranes during endocytosis [12,13], while NA is required for viral release from infected host cells, and it acts as a receptor destroying enzyme, cleaving terminal sialic acid residues from glycoproteins present at the cell surface [14,15,16]. The matrix 2 (M2) protein is also found in the viral membrane, although in much lower abundance than HA or NA glycoproteins. M2 is a small transmembrane protein that forms a proton-selective ion channel in the viral envelope. M2 promotes uncoating of the vRNPs after membrane fusion and the protein has also an essential role in viral assembly and release [17]. Under the viral envelop, there is an inner shell composed of the matrix 1 (M1) protein, which interacts in the virion with the vRNP and the HA and NA proteins. M1 apart from being a membrane-associated scaffold factor of the virion, acts as a crucial factor for different viral processes during infection, including virion assembly and budding [18,19,20]. The nonstructural (NS) gene or segment 8 of IAV encodes an mRNA transcript that is alternatively spliced to express two viral proteins, the nonstructural protein 1 (NS1), produced from a continuous primary transcript, and the nuclear export protein (NEP), which is produced by an alternatively processed transcript, using a weak 5′ splice site. NEP is also located in the virion and may interact with M1 in the viral particle [21,22,23] (Figure 1A). During the infection, NEP is responsible for the nuclear export of synthetized vRNP, ensuring that the vRNPs are available for packaging [24]. Moreover, NEP has also other functions during IAV infection, contributing to viral budding and to regulate viral RNA synthesis. NS1 is a multifunctional protein and a key viral factor that counteracts the host antiviral responses. NS1 has been shown to inhibit the production of interferon (IFN), the activity and expression of multiple interferon-induced genes (ISG) and the processing and nuclear transport of host mRNAs causing cellular shut-off [25,26]. Segment 3 of IAV also encodes two proteins, the polymerase component PA and PA-X. PA is translated directly from the PA mRNA, whereas PA-X is translated using a +1 frameshift mechanism from the same open reading frame (ORF) [9]. Synergistically with NS1, PA-X is also able to block the cellular antiviral responses by inhibiting host protein expression. Moreover, the PA-X protein has been shown to modulate host inflammation, immune responses, apoptosis, and virus pathogenesis [25,26,27,28,29,30]. 

### 1.2. Influenza Virus Importance in Human Health

Human IAV infections cause contagious respiratory diseases associated with mild to severe respiratory illness or even death, and they are considered as an important public health threat worldwide, which also results in significant economic losses [31,32,33]. IAV are divided into multiple subtypes, based on the HA and NA glycoproteins. Currently, there are 18 HA (H1 to H18) and 11 NA (N1 to N11), but the growing IAV surveillance programs and sequencing technologies could increase the number of subtypes in the following years. IAV can infect a wide range of avian and mammalian species, although the natural reservoirs of IAV are shorebirds and wild waterfowls [34,35,36,37]. Among all the HA and NA subtypes, only H3N2 and H1N1 IAV subtypes are circulating in human beings and they are responsible for annual recurrent epidemics that affect the entire world [38,39]. Seasonal influenza infections are prevented and controlled through annual vaccination campaigns to decrease IAV infections and viral transmission as well as to reduce their negative impact in the global economy. However, although vaccination remains the most effective approach to protect the population from seasonal infections, the effectiveness of current vaccination approaches is suboptimal [16,31,32,33,39,40,41,42,43,44]. Thus, the production of improved prophylactic approaches, including universal vaccines, are highly desired. Concerns associated with IAV are further aggravated by the adaptive capacity of the viruses to infect new hosts or escape to the immune system, as well as their ability to transmit efficiently in the population and the limited therapeutic options to treat viral infections [14,16,25,45].

Because of the ability of IAV to modify their genome using two main evolutionary mechanisms, antigenic drift and shift, viruses encoding novel antigenic proteins to which the population has limited or no preexisting immunity can be generated [10,31,37,40]. For that reason, seasonal vaccines have to be reformulated yearly to guarantee that the viral glycoproteins (HA and NA) in the vaccine match seasonal viruses circulating worldwide [38,43,46]. In addition, IAV variability can lead to the generation of new virus strains with pandemic potential. For example, the first IAV pandemic of this century occurred in 2009 and it is estimated that in approximately one year, the pandemic 2009 H1N1 (pH1N1) IAV infected more than 600,000 human beings, causing near 16,000 deaths in over 200 countries [40,41]. In addition, although only H1N1 and H3N2 are circulating in humans, the avian H5, H7, and H9 subtypes eventually cross the species barrier to infect humans, representing a new and serious public health problem [13,37,47,48,49].

### 1.3. Innate Immunity in IAV Infections

The cellular defense mechanisms provided by the innate immune system are a formidable barrier to inhibit virus infections [50] and involve the recognition of pathogen-associated molecular patterns (PAMPs) by pattern recognition receptors (PRRs). This recognition leads to the activation of signaling pathways and the production and secretion of IFNs of type I (IFNα and IFNβ) and III (IFNλ2 or IL-28A, IFNλ3 or IL-28B, and IFNλ1 or IL-29), and chemokines and cytokines involved in inflammatory processes [50]. IAV RNAs are mainly recognized by the endosomal, membrane-associated PRR Toll-like receptors (TLRs) 3 (double-stranded RNAs, dsRNAs) or 7/8 (ssRNAs), respectively [50,51], by the cytoplasmic PRR retinoic acid-inducible gene I (RIG-I), which detects dsRNA and 5′- triphosphates of the negative ssRNA viral genome [50,52], generated during replication of multiple viruses, by the NOD-like receptor family member NOD-, LRR- and pyrin domain-containing 3 (NLRP3), which recognizes various stimuli (see below) [53] and by the absent in melanoma 2 (AIM2) protein, recognizing not well-characterized influenza stimuli [54]. The result of PRR detection of viral PAMPs is the activation of multiple transcription factors, such as the nuclear factor kappa β (NF-κB), the activator protein 1 (AP-1), and IFN regulatory factors (IRF)-3 and IRF-7, which are responsible for the transcription of IFNs [50,55,56] and pro-inflammatory cytokines [57]. 

Secreted type I and III IFNs signal through different receptors in a paracrine or autocrine way to induce the transcription of IFN-stimulated genes (ISGs), several of which counteract viral replication [50,56,58]. Just as an example mentioned below, IFITM3 is an ISG playing antiviral roles against influenza virus infection and other viruses [59]. Type I and III IFNs signaling pathways lead to the post-translational phosphorylation of the signal transducer and activator of transcription (STAT) 1 and 2 transcription factors [60], being the tyrosine kinase 2 (TYK2) and Janus protein tyrosine kinase 1 (JAK1) critical for the phosphorylation [61]. Moreover, STAT1 is phosphorylated by IKKε during IFN signaling and this step is important for the IFN-inducible innate immune response [62,63]. Upon phosphorylation, STAT1 and STAT2 associate with IRF-9 forming the heterotrimeric ISG factor 3 (ISGF3) complex [60]. This heterotrimeric complex then translocates to the nucleus, and binds to IFN-stimulated response elements (ISREs) located in the promoters of ISGs, up-regulating their expression [60,64]. 

Inflammatory cytokines, such as interleukins (IL)-1A IL-1B and tumor necrosis factor (TNF)-α contribute to the proliferation and migration of different immune cells, such as monocytes, macrophages, neutrophils, and natural killer (NK) cells, to the infected tissue. NK cells have the ability to kill virus-infected cells, are important for the activation of a protective cytotoxic T lymphocyte (CTL) response [65], and NK-cell IFN-γ production is augmented by T-cell IL-2 production in recall responses [66]. Neutrophils and resident alveolar macrophages are also important for virus clearance, due to their ability to destroy infected cells [67]. In addition, cytokine signaling improves dendritic cells (DC) maturation, increasing the induction of adaptive immune responses by antigen presentation and co-stimulation [68,69]. These adaptive immune responses initiated upon innate immune activation are required for protection and viral clearance [70].

NLRP3 is expressed by myeloid cells such as macrophages, monocytes, neutrophils, and dendritic cells [71] or by human bronchial epithelial cells [72]. Upon stimulation, NLRP3 activates the inflammasome system, activating caspase-1 and leading to pro-inflammatory processes through the processing and activation of proIL-1B, proIL-18, and proIL-33 [73]. NLRP3 senses IAV dsRNA [74], and PB1-F2 protein [75]. Furthermore, protein flux through the viral M2 ion channel activity in the trans-Golgi network activates NLRP3, leading to inflammasome activation [76]. In addition to NLRP3 activation, IAV activates the inflammasomes through AIM2, increasing IAV-induced lung injury and mortality [54].

The complement system is an important branch of innate immunity that plays an essential role in the clearance of pathogens. The complement system is triggered by three main pathways, the classical, the lectin, and the alternative pathways [77]. The first two pathways are activated with the help of pattern recognition molecules, whereas the alternative pathway is activated spontaneously. Interestingly, it is known that viruses are recognized by the three pathways. In the classical pathway, the C1 complex recognizes antigen-antibody complexes, which are formed on the pathogen surface. C1QBP (Complement C1q Binding Protein) can bind to the globular heads of C1q molecules, activating the classical pathway [78]. On the other hand, in the lectin pathway, the mannan-binding lectin (MBL)/ficolin/mannan-binding lectin serin protease (MAP) complex recognizes specific carbohydrates on the pathogen surface. Complexes activated after the classical and lectin pathways, cleave C4 and C2, resulting in the generation of C4bC2a (C3 convertase). In the alternative pathway, spontaneous hydrolysis of native C3 results in the formation of C3b-like C3 that binds factor B and after cleavage by factor D forms the initial C3 convertase [77]. The three pathways converge at the cleavage of C3 into C3a and C3b by C3 convertases (C4b, 2a and C3b, Bb). Then, the C3b molecules formed bind covalently to the C3-convertases forming the C5-convertases that cleave C5 into C5a and C5b. CD55 blocks C3 and C5 activation by preventing the formation of new C3 and C5 convertases [79]. C5b starts the formation of C5b-9 or the membrane attack complex (MAC). Next, C8 binds to the membrane attached trimer and begins binding and polymerization of C9 that is inserted into the membrane, inducing virolysis [77].

Unregulated complement activation could play a central role in the acute lung injury (ALI) pathology induced by highly pathogenic viruses, including severe acute respiratory syndrome (SARS) coronavirus and avian IAV H5N1, and H7N9 [80]. In virus-induced acute lung diseases, high levels of chemotactic, and anaphylatoxic C5a can be generated as a result of excessive complement triggering and causing a “cytokine storm”. Accordingly, the blockade of C5a signaling has been involved in treating the ALI induced by highly pathogenic viruses [80]. 

### 1.4. Single Nucleotide Polymorphisms (SNPs)

**Figure 2:**
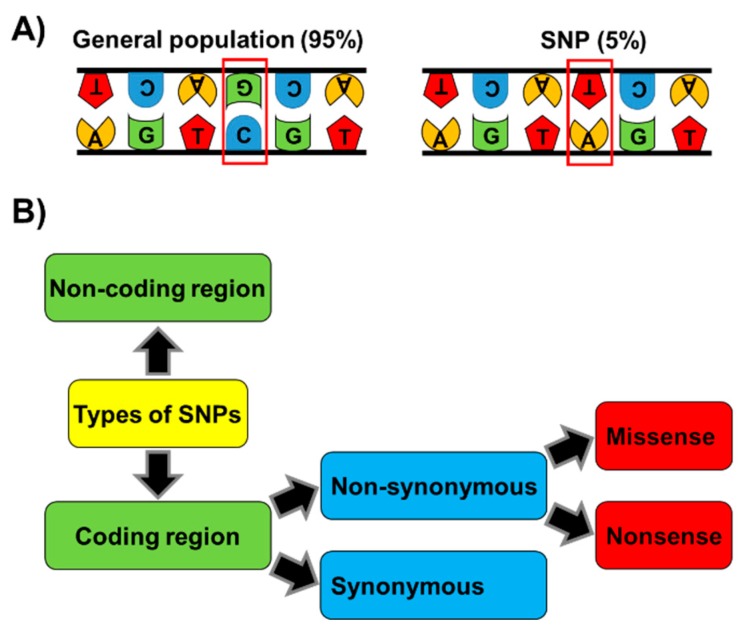
**Single nucleotide polymorphisms (SNPs).** (**A**) An SNP is a variation on a single nucleotide which may occur at some specific point in the genome and that causes variations in DNA sequences between members of the same species. (**B**) Types of SNPs: DNA variation can be located in non-coding or coding regions. SNPs within a coding sequence can be synonymous if they do not produce an amino acid change (silent mutation), or non-synonymous if they affect the protein sequence. Non-synonymous changes can be divided into missense (producing an amino acid change in the protein) or nonsense (producing a truncated or longer protein).

Currently, particular attention is being paid to single nucleotide polymorphisms (SNPs) that are loci within the genome of an organism in which two or more alleles can exist. SNPs affect a single nucleotide or base pair and they are one of the most frequent types of genetic variations in the genome [81,82,83]. SNPs need to be presented into the population with a frequency equal to or greater than 1% to be considered as polymorphisms. There are multiple types of SNPs, depending on their location that can be in different regions of the genes such as promoters, exons, introns or UTRs (Figure 2). SNPs in coding regions are classified as synonymous, when a nucleotide substitution does not change the amino acid sequence of the encoded protein, although other effects, such as changes in mRNA structure or folding may account for variation in protein expression. On the other hand, non-synonymous SNPs are divided in missense or nonsense. In the first case, nucleotide substitution results in the change of one amino acid for another, affecting the protein sequence coded by a gene and therefore may lead to its dysfunction. In contrast, nonsense mutations are produced when instead of substituting one amino acid for another, the altered gene contains an early stop codon in the ORF or a stop codon is abrogated, producing an elongated protein. This type of mutations results in shortened or elongated proteins leading typically to nonfunctional proteins. The functional role of SNPs in coding areas of the genome can be easily analyzed by studying the gene products. However, most SNPs fall within non-coding genome regions, therefore, predicting their effects is challenging. For example, SNPs in the promoter regions could affect their activity and regulation producing changes in gene expression levels. SNPs in UTRs or intron regions have been related with an effect in protein translation or the production of splice variants of transcripts, leading to longer or shorter protein sequences, respectively. 

In summary, SNPs may influence gene regulation, the structure and stability of RNA, the expression of RNAs or proteins, the conformation and function of proteins, etc. Thus, the identification of SNPs in genes and the analysis of their effects may lead us to better understand gene function or their impact on human health [84]. In fact, SNPs that are or could be important for multiple human pathologies, such as cancer, diabetes, heart disease, schizophrenia, blood-pressure homeostasis, and autoimmune or metabolic diseases, have been identified [85,86,87,88,89,90,91]. Moreover, some described SNPs increase the human susceptibility to getting infected by viruses, bacteria or other pathogens [84,86,92,93,94,95,96,97]. Advanced sequencing and bioinformatics technologies have allowed the identification of a large number of human SNPs whose information is accessible in the databases. Nevertheless, the biological significance and function for most of the SNPs found in the human genome remain unknown. Currently, the scientific community recognizes the importance of this kind of genome variations that can act as biological markers and assist researchers in multiple aspects, such as: (1) Locate genes associated with multiple diseases, (2) anticipate an individual’s response to a specific infection, (3) predict population responses to several treatments such as drugs or vaccines, (4) design individualized therapies, (5) identify markers for medical testing, (6) perform pharmacogenetic studies, etc. This review focuses on the role of known SNPs on IAV infection, as well as their impact on the effectiveness of vaccines against IAV.

## 2. SNPs in Host Genes Affecting IAV Disease

Risk factors, including underlying co-morbidities, age, and pregnancy, affect IAV susceptibility, but do not explain all the conditions under which serious IAV-associated disease can occur, making likely that SNPs in viral and host genes affect IAV susceptibility and the outcome of the disease. In fact, there are some examples of the presence of SNPs in host genes affecting influenza severity (Table 1), which will be discussed in this review. SNPs affecting IAV disease have been found in genes recognizing viral components, in transcription factors important for IFN production and signaling, in ISGs with antiviral activities, and in genes involved in inflammation.

TLR3 recognizes dsRNA, one of the IAV replication intermediate products, and in turn activates IFN production, leading to an antiviral response. A missense mutation (F303S) of the TLR3 gene was found in one out of three patients developing IAV-associated encephalopathy (IAE), a neurological consequence of severe viral infection [98]. Assays in tissue culture cells showed that a TLR3 receptor encoding the missense F303S mutation was impaired in activating the transcription factor NF-κB, and in triggering downstream signaling via the IFNβ receptor, indicating that this genetic polymorphism could lead to increased IAV replication [98]. In a study of 51 Italian children diagnosed with IAV H1N1 infection, an additional TLR3 SNP (rs5743313, genotype C/T) was identified [99]. This TLR3 SNP was found in all the children developing IAV-associated pneumonia (18 cases). However, the SNP was found in significantly less proportion in children with milder disease, suggesting a link between TLR3 and IAV pathogenicity. Furthermore, in a multicenter study involving 275 adult cases of avian H7N9 and pH1N1 IAV, in mainland China and Hong Kong, the TLR3 CC rs5743313 SNP was associated with fatal cases [100].

In addition to IAV, there are other examples of SNPs in TLR3 or TLR3 signaling genes affecting viral infections. For instance, susceptibility to Chikungunya virus (CHIKV) infection is highly increased in human and mouse cells with defective TLR3 molecules [101]. Furthermore, *TLR3* SNPs, rs3775292, and rs6552950, leading to unknown functional consequences, were associated with an increased risk of CHIKV disease occurrence [101]. Patients with impaired TLR3-mediated responses show an elevated susceptibility to Herpes Simplex-1 Virus (HSV-1)-mediated encephalitis by encoding TLR3-deficient alleles [102,103], or by encoding deficient TRAF3, TBK1 and TRIF molecules, leading to impaired TLR-3 signaling [104,105,106]. In a Saudi Arabian population, the TLR3 rs78726532 SNP was strongly associated with Hepatitis B (HBV) and Hepatitis C (HCV) virus infections when compared to that in healthy control subjects [107,108]. The TLR3 rs5743314 C allele was also associated with HCV-related liver disease progression (cirrhosis and hepatocellular carcinoma) [107]. However, the functional effects of these SNPs seem to be unknown.

RIG-I detects dsRNA and 5′-triphosphates of the negative ssRNA IAV genome, leading to innate immune responses activation [52]. A Caucasian male patient with severe IAV H1N1 infection during the 2009 swine flu pandemic showed two heterozygous variants (one in each chromosome): p.R71H (SNP rs72710678) and p.P885S (SNP rs138425677), located, respectively, in the caspase activation and recruitment domain (CARD) and RNA binding domains of RIG-I [109]. These variants significantly decreased the recognition function of RIG-I, and therefore, patient cells proved impaired antiviral responses to RIG-I ligands and elevated proinflammatory responses to IAV, providing evidence for dysregulation of the innate immune response and increased immunopathology [109]. These results suggest that these RIG-I polymorphisms may have contributed to severe IAV outcome in this patient and reinforce that RIG-I variants should be evaluated in future studies of host factors affecting ssRNA virus infections.

IRF-7 is a transcription factor that increases interferon (IFN) production in response to viruses [110,111,112]. A patient suffering from an unusual life-threatening disease after pH1N1 infection encodes homozygous null mutations in the IRF-7 factor. Both IRF-7 alleles from this patient encode mutations c.1228T>G/T (F410V) and c.1261C>T/C (Q421X), which are mutations decreasing the ability of IRF-7 to induce the transcription of IFN genes after IAV infections [113]. These findings suggest that IRF-7-dependent production of type I and III IFNs is required for controlling IAV infections in humans. The rare allele A of two IRF-7 SNPs, rs12272434 and rs12290989, both located at exon/intron boundaries, were significantly associated with impaired levels of IFNα production by human plasmacytoid dendritic cells (pDCs) in response to human immunodeficiency virus 1 (HIV-1) infection [114]. Therefore, these polymorphisms may affect the ability of human subjects to control HIV-1 infections, reinforcing the role of IRF-7 in controlling viral infections. However, the effect of these SNPs should be further studied. 

IRF-9 is a transcription factor essential for IFN signaling and the transcriptional induction of ISGs [60]. STAT1 and STAT2, when phosphorylated, associate with IRF-9 to form a heterotrimeric ISG factor 3 (ISGF3) complex [60], which translocates to the nucleus, and binds ISREs present in the promoters of ISGs, up-regulating their transcription [60,64]. A homozygous, loss-of-function mutation in IRF-9 was described in a child born to first-cousin Algerian parents and living in France affected by a severe pulmonary influenza infection [115]. In particular, the homozygous mutation (c.991G>A) occurred in the final nucleotide of exon 7 and disrupted the essential splice site at the boundary of exon 7 and intron 7, leading to deleted IRF-9 proteins. The consequence of this mutation was an impaired activation of IRF-9, and therefore, an impaired transcription of ISGs, many of which show antiviral activities [115]. Similarly, a family in which several members showed a surprising susceptibility to infection by different viruses, including IAV, also showed to be IRF9 deficient [116]. The index patient, a boy with 10 years born at term from healthy consanguineous parents (first cousins of Portuguese origin and residing in Venezuela) encoded a homozygous splicing mutation in the IRF9 gene. The mutation, c.577+1G>T, was located in the donor splice site of introns 5 and 6, leading to transcripts lacking exon 5. IRF9 protein expression was undetectable in cells transfected with the c.577+1G>T IRF9 construct, suggesting that either the protein was quickly degraded or the mRNA was not translated. Again, IRF9-deficient cells showed a profound defect in inducing the expression of multiple ISGs [116]. Collectively, these findings show that human IRF9- and ISGF3-dependent type I and III IFN responsive pathways are essential for controlling viral infections, including IAV.

The antiviral protein IFITM3 is an ISG which abrogates the release of IAV content from late endosomes into the cytoplasm [59]. In addition, IFITM3 promotes the survival of mouse lung-resident CD8+ T cells following IAV challenge, which may help clear the infection [117]. Furthermore, mice in which the expression of IFITM3 is abolished, showed severe disease after IAV infection, compared to parental mice [118]. One of the clearest associations of SNPs in genes affecting influenza severity is located in the ISG IFITM3. The human IFITM3 gene is encoded by two exons and is predicted to encode two splice variants that differ in the first amino-terminal 21 amino acids. Different studies have described the effect of IFITM3 SNPs in influenza disease severity. Northern European patients infected with IAV pH1N1 2009 virus requiring hospitalization showed over-representation of the SNP rs12252 in the IFITM3 gene, in which the majority T allele is replaced for a minority C allele [118]. This leads to an alteration of the first splice acceptor site, originating an IFITM3 protein lacking the first 21 amino acids (NΔ21) due to the protein starting from an alternative start codon. According to these results suggesting that this SNP could affect influenza disease, the minority (CC) variant rendered homozygous cells more susceptible to IAV infection, and this susceptibility correlated with decreased levels of IFITM3 protein expression in comparison to the majority (TT) variant cells [118]. Furthermore, cells expressing the NΔ21 protein showed an impaired ability to restrict viral replication when compared to wild-type IFITM3 cells [118]. This data is consistent with previous results which show that the amino-terminal 21 amino acids of IFITM3 are relevant for attenuating vesicular stomatitis virus (VSV) replication in vitro [119]. Moreover, the CC genotype was found in 25% of Chinese patients showing mild disease after pH1N1 virus infection compared to 69% in patients developing a severe pH1N1 virus infection. In addition, the CC genotype was estimated to confer a six-fold increased risk for severe infection than the CT and TT genotypes [120], reinforcing the idea that IFITM3 is a factor affecting human IAV disease [121]. In another study, over-representation of the IFITM3 CC genotype was detected among fatal cases of Chinese patients infected with IAV pH1N1 and H7N9 viruses [100], and in a more general study, including twelve studies published before February 2018 with more than 16,000 subjects, revealed increased risk of severe influenza in both the East Asian and White populations in the subjects encoding the IFITM3 CC genotype [122].

Another important SNP (rs34481144) associated with risk of severe influenza in humans from the United States (US) infected with seasonal IAVs is located in the 5′-UTR of the IFITM3 gene [123,124]. This SNP affected IFITM3 expression being the risk allele associated with lower mRNA expression. The mechanism for this lower mRNA expression involves the decreased IRF-3 binding and increased binding of the transcriptional repressor CCCTC-binding factor (CTCF) in promoter-binding assays for the risk allele [123]. Moreover, the risk allele disrupted a CpG site that becomes differentially methylated in CD8+ T cell subsets, leading to less CD8+ T cells in the airways during natural influenza infection in the carriers of the risk allele, and suggesting that a critical role for IFITM3 may be to promote immune cell persistence at mucosal sites [123]. 

Interleukins 1A and 1B (IL-1A and IL-1B, respectively) are inflammatory cytokines that play critical roles in recruiting immune and inflammatory cells and developing adaptive immune responses. Furthermore, accumulating evidence suggests that both cytokines play central roles in innate immunity against viral infections [125]. The frequencies of SNP (allele C) located 31 base pairs upstream from the transcription start site (rs1143627), on the IL-1B promoter were associated with increased risk of influenza disease in Chinese subjects [126]. This nucleotide change is localized in a TATA-box motif of IL-1B and modulates the transcription activity of IL-1B by binding to multiple transcription factors [127]. The allele T of rs1143627 enhanced IL-1B protein expression, as indicated by several reports [128]. People carrying allele T showed a higher IL-1B expression, which could lead to increased IFNγ production, which promotes virus clearance [129]. In contrast, expression of IL-1B may be decreased in individuals who carry allele C, leading to a weaker immune response during viral infection. In addition, a T allele in IL-1A gene (SNP rs17561) increased the risk of IAV pH1N1 susceptibility, as observed in Chinese subjects [126]. The SNP rs17561 introduces a nonsynonymous mutation (A114S) in IL-1A protein, suggesting that this genetic variant may lead to a functional variation in host susceptibility to pH1N1. Nevertheless, the molecular mechanism needs to be evaluated and the real risk of these alleles should be analyzed in larger populations.

TNF-α is a pro-inflammatory cytokine which orchestrates the host´s defense. A minor allele (A) at position -238 of TNF (SNP rs361525) was more frequent in Greek patients infected with pH1N1 virus compared to control subjects [130], and developing pneumonia was more uncommon in Greek and Mexican subjects with no copies of the minor allele compared to subjects with at least one copy of the minor allele [130,131], leading to the hypothesis that this SNP allele could be linked with an elevated susceptibility to infection with the pH1N1 virus [124,130]. Decreased TNF-α expression was observed in subjects encoding the minor allele at position -238 [92]. This may explain how SNPs leading to lower production of TNF-α may predispose to more severe clinical symptoms following IAV infections. However, the TNF-α rs 1800629 minor A allele, associated with higher levels of TNF-α expression, was associated with susceptibility to Japanese Encephalitis virus infection in an Indian population [132]. The TNF-α rs 1800629 minor A allele was a risk factor to develop liver cirrhosis and hepatocellular carcinoma following HBV infection in a Han Chinese population [133], suggesting that the protective or deleterious roles of TNF-α expression may vary depending on the infecting virus. 

Chemokine receptor 5 (CCR5) is expressed mainly on macrophages, T cells, and dendritic cells. CCR5 mediates leukocyte chemotaxis in response to its ligands, including MIP-1a, MIP-1b, and RANTES. It can help direct multiple immune cell subsets, including regulatory T cells or Th17 cells to sites of infection, supporting the antiviral immune response. Evidence in humans support that homozygosity for the CCR5-Δ32 allele, a naturally occurring polymorphism of CCR5 encoding a 32-bp deletion, prevents its expression on the cell surface, and is linked with an elevated susceptibility to West Nile virus (WNV) [134] and with increased severity of illness among patients infected with pH1N1 [135], although this evidence is modest due to the limited number of subjects analyzed. In contrast, homozygous carriers of the Δ32 mutation are resistant to HIV-1 infection because this molecule, absent in the cell surface in subjects encoding the deletion, is a molecule normally used by HIV-1 to enter CD4+ T cells [136].

CD55 is an important complement regulatory protein which blocks C3 and C5 activation by preventing the formation of new C3 and C5 convertases, two proteases involved in inflammation and complement activation. Consequently, CD55 protects cells from complement attack and decreases amplification of the complement cascade [79]. The CD55 SNP (rs2564978, genotype T/T) was significantly associated with severe IAV infection in Chinese patients infected with pH1N1 2009 virus [137] and was associated with increased death risk in Greek patients [138]. The rs2564978 SNP of CD55 is located in the minimal promoter region [139] and individuals with this genotype showed significantly lower levels of CD55 expression in comparison to those with the more frequent allele [137]. Therefore, patients who carry the T/T genotype may have more robust complement activation during IAV infection, resulting in enhanced inflammation and disease severity [47,79]. According to these results, the polymorphism rs2564978 in gene CD55 was linked to disease severity in adult Chinese cases of avian (H7N9) and human pH1N1 IAV in another study [100]. However, these findings need to be confirmed in bigger cohorts. 

C1QBP can bind to the globular heads of C1q molecules, activating the classical pathway of complement [78]. An increased risk of severe disease after IAV infection was found in patients homozygous for the minor allele of the SNP rs3786054 in European and Mexican populations [138,140]. However, the effect of this SNPs on gene expression and function is undescribed.

Soluble pattern-recognition molecules, forming part of the innate immune system, can neutralize IAV infection. Particularly, the serum mannose-binding lectin (MBL), several secreted human C-type lectins of the collectin family, collectin 11, and the pulmonary surfactant proteins (SP) –A1, –A2, and –D (SFTPA1, SFTPA2, and SFTPD, respectively), may neutralize IAV infectivity in vitro [141]. Mice lacking SP-A or SP-D were more susceptible to IAV infection, indicating that SPs exert relevant roles against IAV infection [142,143,144]. Two frequent SP-A2 (SFTPA2) missense alleles (rs1965708-C, leading to the mutation Q223K and rs1059046-A, leading to the mutation T9N) were associated with acute respiratory failure, mechanical ventilation, and acute respiratory distress syndrome after infection with pH1N1 2009 virus in a Spanish population [145]. 

In addition to C-type lectins, S-type lectins have been described, such as galectins, which recognize galactose-containing oligosaccharides present in the cellular plasma membranes and in viruses, such as IAV. Importantly, intranasal treatment of galectin-1 enhanced survival of mice infected with IAV by reducing viral load, apoptosis, and inflammation in the lung [146]. Moreover, galectin-1 knockout mice showed increased susceptibility to influenza virus infection than wild-type mice [146]. To study human genetic susceptibility to avian IAV H7N9 infection, a genome-wide association study involving 106 heavily-exposed healthy poultry Chinese workers and 102 IAV H7N9 patients was performed [147]. Functional variants of galectin-1 gene, including rs4820294 and rs13057866, causing increased expression levels of galectin-1 expression, may confer more protection from IAV H7N9 infection to the carriers of these variants [147]. 

The cleavage of the IAV HA by host proteases is critical for viral infectivity. TMPRSS2 is a type II transmembrane serine protease family member, which was shown to activate HA proteins of multiple human IAVs in tissue culture cells. Furthermore, deletion of Tmprss2 in mice impairs the spread of H1N1 influenza viruses, including the pH1N1 2009 swine IAV [148]. In addition, bodyweight loss and survival after H3N2 IAV infection were less severe in Tmprss2 mutant mice compared to wild type mice [148]. The genetic predisposition to severe pH1N1 2009 influenza virus was evaluated in Chinese human subjects, finding that the GG genotype of rs2070788, leading to increased expression of TMPRSS2, was a risk variant to severe pH1N1 influenza [149]. Furthermore, rs2070788 and rs383510, both of them associated with increased gene expression, were significantly associated with the susceptibility to IAV H7N9 [149]. 

## 3. SNPs in Genes that Influence the IAV Vaccine Response

Currently, IAV vaccines are the main strategy to prevent IAV infection, though their effectiveness is suboptimal in many cases. Notably, the efficacy of vaccines against IAV infections can fluctuate and there is a significant immune response variability across the population. Factors such as previous exposure to IAV infections or vaccines, age, and the closeness of the match between the vaccine and circulating strains are important to explain differences in vaccine effectiveness between seasons and group populations [44,46,150,151,152]. However, multiple reports have demonstrated that the host genetic background and polymorphisms on key immune response genes modulate the immune response to infection or vaccination [153,154,155,156,157,158,159,160]. Therefore, new insights into IAV-host interaction and immune response modulating factors could allow us to design better vaccination strategies. 

SNPs may modify the humoral immune response after IAV vaccination. Therefore, their impact on the immune responses induced after IAV vaccination are being analyzed [153,154,155,156]. The major histocompatibility complex (MHC) is localized in chromosome 6 of the human genome, it includes multiple genes and exhibits considerable diversity between populations. Moreover, in this genomic region, there is a higher presence of SNPs than in other sections of the genome. MHC class I and class II molecules have an essential role in the adaptive immune system in response to infections. Both classes of proteins bind peptide fragments derived from pathogens to be presented on the cell surface for recognition by appropriate T cells [97,161,162]. In those genes, the human leukocyte antigens (HLA) class I and II are important because of their role in the immune system. Gelder et al. studied whether HLA class II polymorphisms modulate anti-IAV antibody responses to vaccination in a United Kingdom population [154]. For that, a cohort of HLA-typed donors at risk was investigated, and hemagglutination-inhibition (HAI) titers were evaluated before and 28 days after the administration of seasonal trivalent influenza vaccine. A correlation between HLA class II alleles and IAV HAI titers in the influenza risk group was found. Moreover, a positive association between non-responsiveness to influenza vaccine and HLA-DRB1*07 and a negative association with HLA-DRB1*13 and HLA-DQB1*0603-9/14 [154] was reported, suggesting that polymorphisms in HLA class II molecules affect antibody responses to IAV vaccination. These findings are important because they could potentially identify individuals who may not be protected by current vaccination approaches. 

In another study, Poland et al. analyzed the immunogenetic relationships between HLA, cytokine and cytokine receptor gene polymorphisms in the induction of antibodies in response to inactivated seasonal vaccines [156]. Authors did not find statistically significant associations between HLA class II alleles and IAV HAI titers. However, they established a positive association of some HLA class I alleles and IAV H1N1 HAI titers, including HLA- A*1101, A*6801, B*3503, B*1401, and C*0802. In contrast, they did not find associations between the HLA-A, B or C alleles and HAI antibody titers for IAV H3N2. In addition, when authors evaluated a panel of 586 cytokine and cytokine receptor SNPs, they identified several significant associations between SNPs, in regulatory or coding regions of cytokine (IL-6, IL-12B) or cytokine receptor (IL-1R, IL-10RB, TNFRSF1A) genes and variations in HAI antibody titers for IAV H1N1 [156] (Table 2). Notably, SNPs from three genes, IL-6 (rs1800796), IL-12B (rs3212227) and IL-1R1 (rs3732131) revealed links with IAV H1N1-induced antibody responses in an allele dose-related way. The presence of SNP allele C or G in the IL-12B or IL-1R1genes, respectively resulted in reduced HAI titers. However, high HAI titers in the presence of minor SNP allele G in the IL-6 gene were observed [156]. SNPs associations between cytokine or cytokine receptor genes and IAV H3N2 HAI titers were also identified (Table 2). For example, a variant GA for non-synonymous SNPs within the IL-12 receptor gene (rs2307153; D465G) and TNF receptor 2 gene (rs5746026; K232E) displayed associations with lower HAI titers, while a minor allele T variant (rs12722605) located in the 3′ region of the IL-2 receptor gene was related with high antibody titers (Table 2). These data suggest that host SNPs affect responses to influenza vaccine.

Mannose-binding lectin 2 (MBL-2) is a protein that binds N-acetylglucosamine, mannose, and fucose on different microorganisms and activates the lectin complement pathway [163,164]. Tang et al. studied the presence of SNPs in subjects who received an inactivated influenza vaccine. For that, authors classified the vaccine recipients in poor, normal or adverse responders. They observed that the G to A SNP in the codon 54 allele (rs1800450) in MBL-2 was associated with a decreased risk for the development of adverse or poor responses (Table 2) [165]. In addition, they did not find a significant association between responses and either TNF-α or IL-10 promoter SNPs among the 3 response groups [165]. 

Other SNPs that are not related with immune responses have been also linked to vaccine effectiveness. Egli et al. revealed that the presence of the T/G or G/G genotype (rs8099917, minor-allele) in IL-28B (IFNλ3), a type III IFN, was linked with increased seroconversion in recipients of an inactivated influenza vaccine (Table 2) [153]. Moreover, IAV-stimulated B- and T-cells from the minor-allele carriers exhibited increased HLA-DR and IL-4 expression, respectively. In addition, the expression of IL-28B, but not IL-28A or IL-29, mRNAs was significantly reduced in the rs8099917, minor-allele carriers. Authors also reported that the IL-28B rs8099917 polymorphism affected humoral responses to the IAV vaccine, and had a strong outcome on cellular immune responses by modulating the Th1/Th2 cytokine response [153]. These findings are important because they will help to predict which individuals could not be protected by present vaccines and they can also be used to design personalized vaccine strategies to optimize the immune reaction.

## 4. Conclusions

The sequencing of the human genome together with the development of novel bioinformatic tools have made possible the identification of multiple SNPs. More information is available for the scientific community in the databases. In addition, the identification and study of the human genome variability has opened the opportunity to investigate their association with the risk of developing multiple human diseases facilitating their diagnosis or the susceptibility to infections caused by viruses or other pathogens. Moreover, the knowledge and analysis of genomic variability will be a valuable tool to predict the outcome of prophylactic or therapeutic interventions, including vaccines and drugs. The analysis of human SNPs and their association with IAV infections or vaccination outcomes have just begun. However, current research and data reflect the importance to obtain a better understanding of these relations and the mechanisms underlying the effect of SNPs in the human immune system. In the future, this knowledge could be used to better understand host factors affecting viral replication and disease severity and to develop new and more effective therapeutic strategies against viral infections. 

## Figures and Tables

**Table 1:** Single nucleotide polymorphisms associated with susceptibility and severity of influenza infections.

Gene	Function	SNPs (Type)	References
TLR-3	Recognizes dsRNA, triggering IFN production.	rs not annotated; F303S (NonSyn). rs5743313 (NCR).	[98] [99,100]
RIG-I	Detects dsRNA and 5′- triphosphates of the negative ssRNA IAV genome, leading to innate immune responses activation.	rs72710678; R71H (NonSyn). rs138425677; P885S (NonSyn).	[109]
IRF-7	Transcription factor that increases IFN production in response to viruses.	rs786205223; F410V (NonSyn) rs375323253; Q421X (NonSyn)	[113]
IRF-9	Transcription factor essential for IFN signaling and the transcriptional induction of ISGs.	c.991G>A occurred in the final nucleotide of exon 7 and disrupted the essential splice site at the boundary of exon 7 and intron 7 (NonSyn). c.577+1G>T, was localized in the donor splice site of introns 5 and 6 and led to transcripts lacking exon 5 (NonSyn).	[115] [116]
IFITM3	ISG which abrogates the release of IAV content from late endosomes into the cytoplasm. IFITM3 increases the survival of mouse lung-resident CD8^+^ T cells after IAV infection, which can help clear the infection.	rs12252, leading to an alteration of the first splice acceptor site, leading to an IFITM3 protein lacking the first 21 amino acids (NonSyn). rs34481144, is located in the 5′-UTR and affects IFITM3 expression with the risk allele showing lower mRNA expression (NCR).	[100,118,120,122] [123]
IL-1B	Inflammatory cytokine involved in the development of adaptive immune responses. Furthermore, accumulating data has suggested that IL-1A and IL-1B have critical roles in innate immunity against viral infections.	rs1143627, located 31 base pairs upstream from the transcription start site, on the IL-1B promoter. This nucleotide change is located in a TATA-box motif of IL-1B, affecting the transcription activity of IL-1B (NCR).	[128,129]
IL-1A	Inflammatory cytokine that plays important roles in the development of adaptive immune responses. Moreover, multiple pieces of evidence have suggested that IL-1A and IL-1B play relevant roles in innate immunity against viral infections.	rs17561; A114S (NonSyn).	[126]
TNF-α	Pro-inflammatory cytokine which orchestrates the host´s defense.	rs361525, a minor allele (A) at position 238 (NCR).	[92,130,131]
CCR5	Cytokine receptor which has a role in mediating leukocyte migration in response to its ligands, including MIP-1a, MIP-1b, and RANTES. Furthermore, it can help direct many immune cell subsets, including regulatory T cells and Th17 cells to sites of infection, supporting the antiviral immune response.	CCR5-Δ32 allele (NonSyn).	[135]
CD55	Blocks C3 and C5 activation by inhibiting the formation of new C3 and C5 convertases, which are two proteases involved in complement activation and inflammation. CD55 functions to protect cells from complement attack and decreases the amplification of the complement cascade	rs2564978, resides in the minimal promoter region, affecting gene expression (NCR).	[47,79,100,137,138]
C1QBP	Binds to the globular heads of C1q molecules activating the classical pathway of complement.	rs3786054, localized in an intron (NCR).	[138,140]
SFTPA2	Soluble pattern-recognition molecule that may neutralize IAV infection.	-rs1965708; Q223K (NonSyn). -rs1059046; T9N (NonSyn).	[145]
Galectin-1	Recognizes galactose-containing oligosaccharides present in the cellular plasma membranes and in viruses, such as IAV.	-rs4820294 (NCR). -rs13057866 (NCR).	[147]
TMPRSS2	Type II transmembrane serine protease family member which activates HA proteins of diverse human IAV in tissue culture cells. Deletion of *Tmprss2* in mice impairs the spread of H1N1 influenza viruses, including the pH1N1. Moreover, body weight loss and survival were less severe in *Tmprss2* mutant mice compared to wild type mice after infection with H3N2 IAV.	-rs2070788, localized in an intron (NCR). -rs383510, localized in an intron (NCR).	[149]

Syn-synonymous, NonSyn-nonsynonymous, NCR-non-coding region (Intron, regulatory regions, promoter or UTR).

**Table 2:** Associations between SNPs and IAV vaccine responses.

Gene	Function	SNPs (Type)	Vaccine	Reference
IL-6	Cytokine expressed as a response to infections or tissue injuries. It plays an important role in host defense through the stimulation of acute-phase responses.	-rs1800796 (NCR). - rs2069861 (NCR).	IIV	[156]
IL-12B	Cytokine that serves as a crucial inducer of Th1 cell development.	rs3212227, located in 3´UTR (NCR).	IIV	[156]
IFN-B1	Cytokine released as part of the innate immune response against infection by viruses or other pathogens.	rs1364613 (NCR).	IIV	[156]
TNFRSF1A	Cytokine receptor, its interaction with TNF-α control cell survival, apoptosis, and inflammation.	rs4149621 (NCR).	IIV	[156]
IL-1R1	Cytokine receptor involved in inflammatory and immune responses.	rs3732131, located in 3´UTR (NCR).	IIV	[156]
IL-10RB	Cytokine receptor that mediates the activation of the JAK/STAT signaling pathway leading to the expression of ISG.	rs3171425, located in 3´UTR (NCR).	IIV	[156]
IL-2RA	This cytokine receptor is important for the signaling pathway leading to immune cell differentiation and function.	-rs2228150 (Syn). -rs12722605 (NCR).	IIV	[156]
IL-10RA	Cytokine receptor that is involved in the inhibition of the synthesis of several proinflammatory cytokines.	-rs4252249 (Syn) -rs4252243 (NCR).	IIV	[156]
IL-12RB2	Cytokine receptor that plays a role in Th1 cell differentiation.	rs2307153; D465G (NonSyn).	IIV	[156]
IL-1RN	Cytokine receptor which modulates a variety of immune and inflammatory responses related with IL-1.	-rs315952 (Syn). -rs315951 located in 3´UTR (NCR).	IIV	[156]
TNFRSF1B	Cytokine receptor involved in the recruitment of anti-apoptotic proteins.	rs5746026; K232E (NonSyn)	IIV	[156]
MBL-2	This calcium-dependent protein that plays an important role in innate immunity, and activates the lectin complement pathway.	rs1800450; G54D (NonSyn)	IIV	[165]
IL-28B (IFNL3)	Type III IFN molecule, with brad functions in antiviral responses	rs8099917 (NCR).	IIV	[153]

Syn-synonymous, NonSyn-nonsynonymous, NCR-non-coding region (Intron, regulatory regions, promoter or UTR). IIV: inactivated seasonal vaccine. IL-6, interleukin 6. IL-12B, interleukin 12. IFN-B1, interferon beta 1 (IFNβ). TNFRSF1A, TNF receptor superfamily member 1A. IL-1R1, interleukin 1 receptor type 1. IL-10RB, interleukin 10 receptor subunit beta. IL-2RA, interleukin 2 receptor subunit alpha. IL-10RA, interleukin 10 receptor subunit alpha. IL-12RB2, interleukin 12 receptor subunit beta 2. IL-1RN, interleukin 1 receptor antagonist. TNFRSF1B, TNF receptor superfamily member 1B. MBL-2, mannose binding lectin 2. IL-28 B or IFNL3, interferon lambda 3.
